# Oral bioavailability of microdoses and therapeutic doses of midazolam as a 2-dimensionally printed orodispersible film in healthy volunteers

**DOI:** 10.1007/s00228-022-03406-y

**Published:** 2022-10-18

**Authors:** Mareile H. Breithaupt, Evelyn Krohmer, Lenka Taylor, Eva Koerner, Torsten Hoppe-Tichy, Juergen Burhenne, Kathrin I. Foerster, Markus Dachtler, Gerald Huber, Rakesh Venkatesh, Karin Eggenreich, David Czock, Gerd Mikus, Antje Blank, Walter E. Haefeli

**Affiliations:** 1 Department of Clinical Pharmacology and Pharmacoepidemiology, Heidelberg University Hospital, Im Neuenheimer Feld 410, 69120 Heidelberg, Germany; 2 Hospital Pharmacy, Heidelberg University Hospital, Im Neuenheimer Feld 670, 69120 Heidelberg, Germany; 3 Gen-Plus GmbH & Co KG, Staffelseestrasse 6, 81379 Munich, Germany; 4 DiHeSys, DiHeSys Digital Health Systems GmbH, Marie-Curie-Str. 19, 73529 Schwäbisch Gmünd, Germany

**Keywords:** Printing, Two-dimensional 2D, Inkjet printing, Orodispersible films, Bioequivalence, Midazolam, Healthy volunteers

## Abstract

**Purpose:**

The use of two-dimensional (2D) printing technologies of drugs on orodispersible films (ODF) can promote dose individualization and facilitate drug delivery in vulnerable patients, including children. We investigated midazolam pharmacokinetics after the administration of 2D-printed ODF.

**Methods:**

Midazolam doses of 0.03 and 3 mg were printed on an ODF using a 2D drug printer. We investigated the bioavailability of the two midazolam doses with ODF swallowed immediately (ODF-IS) or delayed after 2 min (ODF-DS) by comparing their pharmacokinetics with intravenous and oral midazolam solution in 12 healthy volunteers.

**Results:**

The relative bioavailability of ODF-IS 0.03 mg was 102% (90% confidence interval: 89.4–116) compared to oral solution and for 3 mg 101% (86.8–116). *C*_max_ of ODF-IS 0.03 mg was 95.5% (83.2–110) compared to oral solution and 94.3% (78.2–114) after 3 mg. Absolute bioavailability of ODF-IS 0.03 mg was 24.9% (21.2–29.2) and for 3 mg 28.1% (23.4–33.8) (oral solution: 0.03 mg: 24.4% (22.0–27.1); 3 mg: 28.0% (25.0–31.2)). Absolute bioavailability of ODF-DS was significantly larger than for ODF-IS (0.03 mg: 61.4%; 3 mg: 44.1%; both *p* < 0.0001).

**Conclusion:**

This trial demonstrates the tolerability and unchanged bioavailability of midazolam printed on ODF over a 100-fold dose range, proving the suitability of ODF for dose individualization. Midazolam ODF-IS AUC_0–∞_ in both doses was bioequivalent to the administration of an oral solution. However, *C*_max_ of the therapeutic dose of ODF-IS missed bioequivalence by a clinically not relevant extent. Prolonged mucosal exposure increased bioavailability. (Trial Registration EudraCT: 2020–003984-24, August 10, 2020).

## Introduction

The broad use of medicines in very diverse and often vulnerable populations such as older patients or children, the substantial variability of drug clearance (e.g., by drug interactions) and thus individual dose requirements, and the development of targeted medicines with a narrow therapeutic index require flexibly adaptable and precise dosing regimens for successful and safe drug therapy. Within days, dose requirements can change by several orders of magnitude [1, 2], making the provision of suitable doses technologically difficult and challenging, and handling and dose adjustment of currently available drugs by health professionals or patients can become complex and error-prone [3]. Therefore, new practicable and low-error technologies are needed that help to adjust the dose safely and reliably to the individual needs of the patient.

Currently, most oral dosage forms are solid, but it is often difficult for vulnerable patient groups, such as older patients and young children, to swallow them [4–7]. In addition, pediatric drug therapy is further complicated because dosing depends on body weight and the changing maturity of elimination organs [8]. Therefore, in order to treat children appropriately, the dosage forms of medicines on the market must often be individually modified, divided, or diluted to obtain the required dose, which frequently leads to medication errors [9–11]. Currently, available solid oral dosage forms are mainly tablets and capsules, while orodispersible tablets would be easier to swallow but are rare, and their current doses cannot be adjusted.

In recent years, several steps have been taken to develop personalized oral dosage forms that meet the challenges of variable dose requirements while facilitating the administration process. One solution may be the use of 2-dimensional (2D) inkjet printing technologies on orodispersible films (ODF) to overcome the challenges of oral drug administration [12, 13]. Digital printing of medicines using 2D/3D print technology has been described in the literature [14, 15]. In this manufacturing process, active pharmaceutical ingredients (API) dissolved in a carrier solution (ink) are printed on polymeric ODF [12, 16]. This dosage form has several advantages: (i) different APIs can be applied to the same ODF; (ii) it is possible to print the API on the ODF in divided, well-separated doses so that the dose can be swallowed whole or changed over a wide dose range by cutting the ODF (dosing flexibility); (iii) mucosal administration can accelerate absorption and increase the bioavailability of certain drugs (including midazolam [17]); (iv) ODF are easier to swallow and their handling is well accepted [18].

Midazolam is a sedative frequently used in children before interventions and—at therapeutic and microdoses—a marker substrate of cytochrome P450 (CYP) 3A4 activity [19, 20]. The oromucosal residence time of midazolam determines how much of the drug will bypass the first-pass elimination, thus increasing bioavailability from 30 to 70% (AUC_0–∞_) [17]. In healthy volunteers, we investigated the bioequivalence, absolute bioavailability, and absorption characteristics of two 100-fold different midazolam doses printed on ODF. Therefore, a therapeutic dose (3 mg) and a microdose of midazolam (30 µg) were printed on a single ODF, and the two doses were separated by a perforated line.

## Methods

### Clinical trial

This was a single-center, open-label, fixed-sequence, phase I clinical trial in 12 healthy volunteers, which was started after approval by the competent regulatory authority (Bundesinstitut für Arzneimittel- und Medizinprodukte, BfArM, EudraCT 2020–003984-24, registration date 10 August 2020) and favorable assessment by the responsible Ethics Committee of the Medical Faculty of Heidelberg University (AFmo-012/2021). All procedures were carried out in accordance with the good clinical practice guideline, the pertinent version of the Declaration of Helsinki, and all specific legal requirements in Germany. The study was conducted between 12 April 2021 and 09 July 2021 at the Early Clinical Trial Unit of the Department of Clinical Pharmacology and Pharmacoepidemiology at Heidelberg University Hospital, which is certified according to DIN EN ISO9001. After having received detailed written and oral information, participants (aged 18–50 years) had to give their written informed consent prior to any study-related procedures.

### Investigational medicinal product (IMP)

ODF were manufactured by Gen-Plus GmbH (Munich, Germany) using purified water, absolute ethanol, sorbitol, and Opadry^®^ II white (polyvinyl alcohol, polyethylene glycol, TiO_2_, and talcum). Midazolam-containing ink was also manufactured at Gen-Plus GmbH and consisted of midazolam hydrochloride (Cambrex Profarmaco Milano S.r.l.; Milano, Italy), purified water, absolute ethanol, and Kollidon^®^ 12 PF (polyvinyl pyrrolidone). Ink containing midazolam was then printed using a 2D printer (Flexdose printer, DiHeSys GmbH, Schwäbisch Gmünd, Germany), which dynamically passes ODF under a stable print head that applies small droplets of ink containing the API and optically measures the size of the individual droplets. The printed ODF were dried on an integrated heating plate, packed air-tight in polypropylene screw top tubes, and stored at room temperature. The ODF contained 3.03 mg midazolam hydrochloride, separated into doses of 3 mg and 30 µg each, corresponding to 2.7 and 0.027 mg midazolam base. The ODF had an area of 10.2 cm^2^ (3.5 × 2.9 cm), and a transverse perforation line divided the rectangular film into an area of about 2.61 cm^2^ containing the 30-µg dose and a larger area (7.54 cm^2^) containing the therapeutic dose. The doses could easily be divided at the perforated line to administer each dose separately.

For comparison, an intravenous (i.v.) microdose of 30 µg midazolam (Dormicum^®^ V 5 mg/5 ml, CHEPLAPHARM Arzneimittel GmbH, Greifswald, Germany), an oral therapeutic dose (as an oral solution) (3 mg Dormicum^®^ diluted in 100 ml of bottle water), and an oral microdose solution (30 µg Dormicum^®^ diluted in 100 ml of bottle water) were administered.

### Study population

Healthy volunteers were screened for underlying diseases using a structured questionnaire, physical examination, electrocardiogram, and routine laboratory, including blood and urine tests, pregnancy tests (females), and screening for illicit drugs. Any intake of drugs or substances known to interact with CYP metabolism, as well as alcohol and grapefruit, were prohibited 2 weeks before and throughout the trial.

### Design and conduct of the trial

**Fig. 1:**
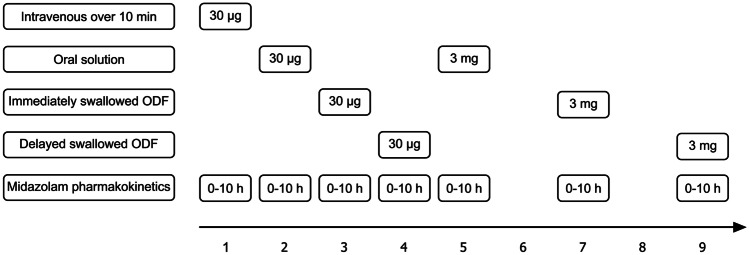
Trial design to evaluate the pharmacokinetics of two different doses of midazolam administered as a short intravenous infusion over 10 min, as oral solution, and as orodispersible films; i.v., intravenous 10 min short infusion, delayed swallowed: after 2 min; ODF, orodispersible film

The trial was designed to assess the absolute and relative bioavailability of different midazolam oral administration forms, the impact of the oral residence time of the ODF, and dose linearity. In a fixed-sequence design, the participants received the following treatments: 30 µg i.v. as a short infusion over 10 min, 30 µg as an oral solution, 30 µg immediately swallowed ODF (ODF-IS), 30 µg delayed swallowed ODF (ODF-DS), 3 mg as an oral solution, 3 mg ODF-IS, and 3 mg ODF-DS (Fig. 1).

Trial visits were separated by a 24 h washout period after the administration of a microdose and 48 h after a therapeutic dose. Participants came to all study visits fasting (> 6 h), and food intake was not allowed until 4 h after midazolam administration. To study the effects of the oral residence time of the ODF on midazolam pharmacokinetics, the ODF was either swallowed immediately followed by 200 ml of bottle water (ODF-IS) without waiting for dissolution, or delayed (ODF-DS), where participants kept the ODF beneath their tongue for exactly 2 min followed by drinking 200 ml of bottled water. After ODF administration, the local tolerance of the ODF was asked and the time after the ODF was reported to have completely dissolved was measured.

Blood samples were collected via a peripheral venous catheter on the forearm before and 5, 10, 20, 30, and 45 min, and at 1, 1.5, 2, 2.5, 3, 4, 5, 6, 8, 10, and 24 h after IMP administration. Participants were monitored for adverse events (AEs) for 10 h after administration and then discharged. Within 14 d after the last IMP administration, they underwent an end-of-treatment examination to confirm their health status. AEs were documented and evaluated for seriousness, relationship to the IMP, and severity based on the US National Cancer Institute’s Common Terminology Criteria for Adverse Events (CTCAE) Version 5.0.

### Analytical procedures

Blood samples for midazolam quantification were centrifuged within 60 min at 2500 g at room temperature for 10 min and frozen in 600 µl aliquots at -20 °C. Midazolam concentrations in plasma were quantified in the department’s analytical chemistry laboratory by tandem mass spectrometry after prior separation by ultra-performance liquid chromatography (UPLC-MS/MS) for samples after administration of 30 µg doses [21] and by high-performance liquid chromatography (HPLC–MS/MS) after 3 mg doses [2]. The assays’ lower limit of quantification was 1 pg/ml for the microdose and 0.5 ng/ml for the therapeutic dose. For microdose midazolam the interday (intraday) precision was < 3.49% (< 5.16%) and the interday (intraday) accuracy ranged from 110 to 112% (105 to 114%). For high-dose midazolam the interday (intraday) precision was < 8.15% (< 12.6%) and the interday (intraday) accuracy ranged from 103 to 104% (94.2 to 109%).

### Statistics

Sample size calculation was conducted based on the assumption of *H*_0_: geometric mean ratio (GMR) of the area under the plasma concentration–time curve extrapolated to infinity (AUC_0–∞_) ≤ 0.8 or ≥ 1.25. In a sample size of 12, a GMR of 1 could be detected with an α-error of 0.05 and a power of 0.82. Standard pharmacokinetics parameters (AUC_0–∞_, peak concentration: *C*_max_, time to reach *C*_max_: *T*_max_, half-life: *t*_1/2_, apparent clearance: Cl/F (dose/AUC_0–∞_), and apparent volume of distribution: Vz/F) were calculated using Phoenix WinNonlin Software 8.2 (Certara, Princeton, NJ, USA) using noncompartmental analyses. Because of the short washout period between the microdose treatments, 9 predose samples showed residual midazolam concentrations. In order to derive a single-dose curve, these concentration profiles were transformed via the reverse superposition principle published by Bauer and co-workers [22]. Statistics were performed and figures were constructed with Prism 9.1.1 (GraphPad Software, La Jolla, CA, USA). According to the guideline on the investigation of bioequivalence of the European Medicines Agency (EMA), the GMR and the 90% confidence interval (CI) of AUC_0–∞_ and *C*_max_ must be within 80–125% to be considered bioequivalent [23]. A one-way ANOVA of log-transformed data was used to analyze the differences between all modes of administration, and a descriptive post hoc Tukey test was performed. A *p*-value < 0.05 was considered statistically significant.

## Results

Twelve healthy volunteers (9 males/3 females) with a median age of 24.0 years (range 22.0–30.8) and a mean (± standard deviation) body mass index of 24.1 ± 5.3 kg/m^2^ were included, and all completed the trial. After ODF administration, the subjectively perceived dissolution time for the 30 µg dose with an ODF area of 2.61 cm^2^ was 30.4 s (20.4–45.3 s) and 55.9 s for the 3 mg dose with an ODF area of 7.54 cm^2^ (38.3–81.6 s, *p* < 0.05), but participants reported difficulty in determining the exact time of complete dissolution. Swallowing was reported to be comfortable after complete dissolution of ODF-DS, except for a bitter taste after therapeutic-dose ODF-DS. When swallowing the ODF immediately, some participants reported that the ODF stuck to the palate and, therefore, swallowing felt uncomfortable.

**Fig. 2:**
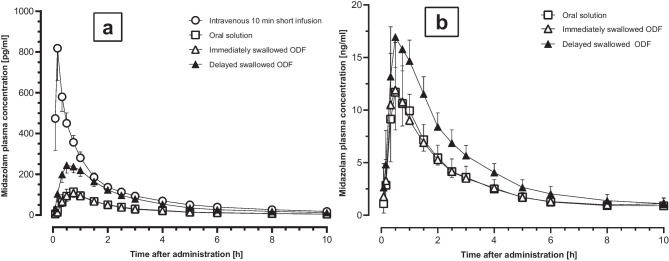
Midazolam plasma concentration–time profile after administration of 30 µg **a** and 3 mg **b** midazolam (geometric mean and 95% confidence interval) to 12 healthy volunteers. ODF, orodispersible film; ODF delayed swallowed: after 2 min

The midazolam plasma concentration–time profiles after administration of a microdose are shown in Fig. 2a and after a therapeutic dose in Fig. 2b, and the corresponding pharmacokinetic parameters are listed in Tables 1 and 2.

**Table 1:** Pharmacokinetic parameters of midazolam in 12 healthy volunteers after administration of two 100-fold different doses in different peroral formulations and one i.v. microdose

Regimen	*C*_max_ [ng/ml]	*T*_max_ [h] median	AUC_0–∞_ [ng/ml]	Absolute *F* [%] GMR (90% CI)	*t*_1/2_ [h]	Cl [l/h]	Vz [l]
Microdose (30 µg)							
Intravenous 10-min short infusion	0.82 (0.66–1.02)	0.17	1.24 (1.11–1.37)	(100)	4.62 (3.71–5.74)	24.3 (21.8–27.0)	162 (133–196)
Oral solution	0.12 (0.10–0.14)	0.75	0.30 (0.25–0.36)	24.4 (22.0–27.1)	3.64 (2.72–4.86)	99.3 (83.4–118)	521 (388–670)
Immediately swallowed ODF	0.12 (0.09–0.15)	0.75	0.31 (0.24–0.40)	24.9 (21.2–29.2)	3.76 (2.66–5.31)	108 (83.4–140)	586 (445–773)
Delayed swallowed ODF*	0.26 (0.22–0.29)	0.63	0.76 (0.65–0.89)	61.4 (56.7–66.6)	3.89 (3.05–4.98)	43.8 (37.3–51.5)	246 (197–307)
Therapeutic dose (3 mg)							
Oral solution	14.5 (11.2–18.7)	0.50	34.6 (30.0–39.9)	28.0 (25.0–31.2)	3.09 (2.40–3.98)	86.7 (75.1–100)	386 (306–488)
Immediately swallowed ODF	13.7 (10.3–18.2)	0.50	34.7 (26.2–46.0)	28.1 (23.4–33.8)	2.88 (2.09–3.96)	95.8 (72.4–127)	398 (312–509)
Delayed swallowed ODF*	18.5 (15.2–22.5)	0.50	54.6 (43.5–68.4)	44.1 (38.8–50.2)	3.24 (2.26–4.66)	61.0 (48.7–76.5)	286 (218–375)

Data are shown as geometric mean (95% CI) unless indicated otherwise

*AUC*_*0–∞*_ area under the plasma concentration–time curve, extrapolated to infinity, *CI* confidence interval, *Cl* apparent clearance (Cl/F except for intravenous infusion), *C*_max_ plasma peak concentration, *F* bioavailability, *GMR* geometric mean ratio, *ODF* orodispersible film, *T*_max_ time to reach C_max_, *t*_*1/2*_ half-life, *Vz* apparent volume of distribution (Vz/F except for intravenous infusion)

*Delayed swallowed ODF were kept for exactly 2 min in the oral cavity and then swallowed

**Table 2:** Geometric mean ratios [%] with 90% CI of AUC_0–∞_ and *C*_max_ of different oral midazolam formulations in 2 different doses

Regimen	Microdose (30 µg)						Therapeutic dose (3 mg)					
	Oral solution		Immediately swallowed ODF		Delayed swallowed ODF*		Oral solution		Immediately swallowed ODF		Delayed swallowed ODF*	
	AUC_0–∞_	*C*_max_	AUC_0–∞_	*C*_max_	AUC_0–∞_	*C*_max_	AUC_0–∞_	*C*_max_	AUC_0–∞_	*C*_max_	AUC_0–∞_	*C*_max_
Intravenous 10-min short infusion	24.4 (22.0–27.1)	14.8 (12.0–18.3)	24.9 (21.2–29.2)	14.2 (10.9–18.5)	61.4 (56.7–66.6)	31.2 (27.0–36.1)	28.0 (25.0–31.2)	17.7 (13.7–22.8)	28.1 (23.4–33.8)	16.7 (12.3–22.5)	44.1 (38.8–50.2)	22.5 (18.4–27.6)
Oral solution			102 (89.4–116)	95.5 (83.2–110)	251 (228–277)	210 (189–234)			100 (86.8–116)	94.3 (78.2–114)	158 (137–181)	127 (108–151)
Immediately swallowed ODF					247 (215–283)	220 (109–255)					157 (133–186)	135 (113–161)

*AUC*_*0–∞*_ area under the concentration–time curve extrapolated to infinity, *C*_*max*_ maximum plasma concentration, *ODF* orodispersible film

*Delayed swallowed ODF were kept for exactly 2 min in the oral cavity and then swallowed. Numerator: column, denominator: row

### Bioavailability

**Fig. 3:**
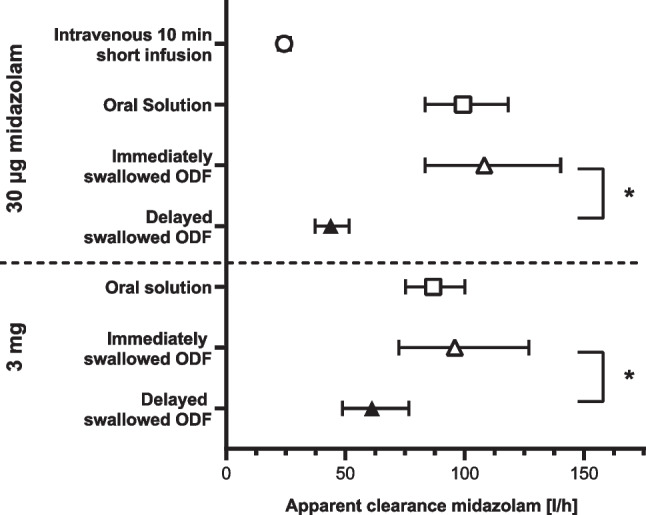
Apparent clearance (dose/AUC_0–∞_) of midazolam after administration of two 100-fold different doses (30 µg and 3 mg) in different dosage forms and modes of administration. *: *p* < 0.0005; ODF: orodispersible film; delayed swallowed ODF: after 2 min. Values are shown as geometric mean with 95% confidence interval

Whereas the elimination *t*_1/2_ of all administration forms and doses was comparable, and the oral bioavailability of ODF-IS was comparable to the corresponding oral administration of midazolam solution (both *p* > 0.9), oral bioavailability increased significantly (+ 147% after the microdose, + 57% after the therapeutic dose, both *p* < 0.001; Table 2) when the ODF had been kept in the oral cavity beneath the tongue for 2 min. At the same time, the corresponding clearance values decreased (Fig. 3).

### Bioequivalence

The microdose administration of midazolam as ODF-IS was bioequivalent to the oral solution (Table 2). After administration of the ODF-IS containing 3 mg midazolam, the AUC_0–∞_ fulfilled the bioequivalence criteria, but the lower limit of the 90% CI of *C*_max_ was slightly outside the bioequivalence range. All other modes of administration showed a significant difference in AUC_0–∞_ and *C*_max_ (*p* < 0.0001). To assess dose proportionality, we compared the dose-normalized AUC_0–∞_ after administration of 3 and 0.03 mg as an ODF-IS, which revealed no statistically significant difference.

### Safety and tolerability

The trial medication and study procedures were well tolerated. In total, 9 AEs occurred, and all were mild (Grade I according to CTCAE 5.0) and transient. The observed AEs were headaches (*n* = 7), diarrhea (1) (assessed as unrelated), and pain at the injection site (1).

## Discussion

Midazolam is a widely used sedative drug, which is approved for use in children of all ages and adults. As a drug that is mainly metabolized by CYP3A4, dose requirements are highly variable and can change rapidly [1]. Therefore, considerable dose flexibility is required, which is not easily achieved with solid oral dosage forms. Moreover, the swallowing of solid oral dosage forms requires a significant degree of cooperation from the patient, which is difficult to achieve in infants or young children but occasionally also in adult patients. In this respect, ODF containing midazolam in printed form may have two major advantages: they can be dosed flexibly and can also be administered to individuals who are not capable or willing of swallowing pills. In this respect, it could also be a suitable dosage form for the treatment of epileptic emergencies.

In this trial, we assessed midazolam exposure of different modes of midazolam administration and two doses to assess bioavailability, bioequivalence to an oral solution, and dose proportionality of a microdose with a therapeutic dose. The bioavailability and overall pharmacokinetics of 2D-printed ODF were similar to earlier reports comparing microdoses with therapeutic doses of midazolam in healthy volunteers [24]. And after administration of the ODF with instant swallowing, midazolam pharmacokinetics was very similar to the pharmacokinetics after administration as an oral solution. Microdoses of midazolam have repeatedly been used to assess drug interactions at the level of CYP3A [20, 25], but the preparation of the microdose requires several dilution steps and can lead to errors. Our findings now suggest that immediately swallowed 2D-printed ODF can also be used to administer midazolam as a CYP3A4 marker in drug interaction trials, which is a formulation that is easy to handle.

All ODF used were well tolerated and handling was easy. The two doses (therapeutic and microdose) were well separated and easy to divide by a perforation line, confirming that different doses can be applied to a single ODF. This may be useful to achieve the dose variety that is otherwise only possible with liquid oral dosage forms. Different doses printed on ODF with a sophisticated and well-thought-out system may be easily divided into the required dose, and among other things, counting of drops is no longer necessary. The ODF dissolved completely and rapidly in saliva in less than one minute. The therapeutic dose required a longer dissolution time (55.9 vs. 30.4 s), which may be related to the larger ODF area of the therapeutic dose (7.54 vs. 2.61 cm^2^).

When immediately swallowed (ODF-IS), midazolam administered as a microdose was bioequivalent to an oral solution. Also, the AUC_0–∞_ of a therapeutic dose was similar, indicating unchanged exposure and complete liberation of midazolam from the ODF. In contrast, the *C*_max_ of the therapeutic dose just missed bioequivalence criteria, possibly because differences in dissolution led to larger variability of absorption. All other pharmacokinetic parameters of midazolam of ODF-IS were comparable and dose-proportional. Theoretically, the differences in dissolution time could easily be circumvented by placing the orodispersible film directly into the drinking glass with which the drug is subsequently swallowed. However, from a clinical point of view, this variation appears to not be relevant.

As expected [17], the bioavailability of midazolam was substantially increased when the ODF was swallowed with a delay. In this trial, we chose an exposure duration with the ODF of 120 s because in a previous trial bioavailability reached its maximum after 100 s of buccal exposure and did not increase further thereafter (up to 150 s) [17], leaving sufficient time for the ODF to disintegrate and the benzodiazepine to be released. Bioavailability increased by > 50%, which is likely due to partial bypass of intestinal and hepatic first-pass metabolism, and *C*_max_ and AUC_0–∞_ increased considerably. *T*_1/2_ remained unchanged with prolonged buccal exposure.

Buccal administration could therefore help to achieve rapid and strong drug effects as desired, for example, before interventions, even when they are to be performed in a fasting state or also in epileptic crises, which are often treated with administration of buccal midazolam, which is faster and easier to administer than many other routes of administration [26]. In addition, an ODF can also facilitate the treatment of patients who are unable, unwilling, or limited in their capacity to swallow or open their mouths, e.g., patients with dysphagia, dementia, or psychiatric diseases.

The limitation of this study is that we examined healthy volunteers in a single age group, as is common for phase I studies. Although this middle age group should be well represented in a future patient population, the intended use for older and very young patients needs to be further evaluated in terms of handling and oral environmental conditions in these age groups. For example, dry mouth could influence the variability of midazolam pharmacokinetics through differential dissolution, and mucosal permeability could be higher in neonates and infants than in older people.

In conclusion, this trial confirmed that midazolam can be printed on ODF and that the new oral formulation makes this benzodiazepine readily absorbable. The immediate swallowing of the ODF provides already exposure comparable to oral midazolam solutions and, by increasing the residence time of the ODF in the oral cavity, oral bioavailability can be substantially increased. 2D-printing on ODF could be a promising novel technology for the oral delivery of flexible doses over a wide dose range (100-fold) with considerable potential to facilitate drug administration in vulnerable patient groups, which are less capable of swallowing and could potentially benefit from this novel technology.

## Data Availability

The dataset generated and analyzed during the current study is available from the corresponding author upon reasonable request.
